# Effects of hazards and sensation‐seeking on intermediate swimming college students’ hazard perceptions

**DOI:** 10.1002/brb3.3338

**Published:** 2023-11-29

**Authors:** Hui Zhang, Li Zi Yun, Shi Luo

**Affiliations:** ^1^ School of Physical Education Hubei Minzu University Enshi City Hubei China; ^2^ School of Physical Education Southwest University Chongqing China

**Keywords:** reaction time, swimmer hazard perception, sensation‐seeking

## Abstract

**Background:**

According to the *Global Report on Drowning: Preventing a Leading Killer* and *Preventing Drowning*: *An Implementation Guide*, two documents released by the World Health Organization in 2014 and 2017, respectively, 372,000 people drown each year globally (approximately 42 per hour), half of whom are below 25 years old. Chinese adolescents aged 18–24 years are the main victim group. Intermediate swimming college students are more susceptible to risk‐taking behavior and drowning while swimming. In particular, college students with high‐sensation‐seeking levels have greater confidence in their swimming ability. Consequently, they tend to overestimate their skills and pursue exciting experiences while underestimating or ignoring the risk factors in the situation. The scores of college students in sensation‐seeking show a significant positive correlation with highly risky swimming behavior. However, the correlation with the reaction time to hazards is unclear. In this study, using previous theories, the sensation‐seeking scale, and the measurement of the reaction time to hazards, we clearly explain why “college students with higher levels of sensation‐seeking have a higher risk of drowning.” We examined the reaction time to hazards and eye movement data of intermediate swimming college students with different sensation‐seeking levels, while controlling the obviousness of hazards in the videos for the experiment.

**Methods:**

We utilized 16 videos of students swimming as experimental stimuli and employed a mixed experimental design of 2 (sensation‐seeking: high, low) × 2 (hazard type: obvious hazard, hidden hazard). Sensation‐seeking, the between‐subjects variable, was categorized into two levels (high and low). The hazard type, the within‐subjects variable, was also divided into two categories (obvious and hidden). We analyzed the disparities in reaction time to hazards and eye‐movement data between intermediate swimming college students with high (*N* = 28) or low (*N* = 28) levels of sensation‐seeking.

**Results:**

Intermediate swimming college students with high levels of sensation‐seeking exhibited significantly longer reaction times to both obvious (*F* = 6.251, *p* = .000 < .001) and hidden (*F* = 49.438, *p* = .000 < .001) hazards compared to their low‐sensation‐seeking counterparts. The first fixation duration of intermediate swimming college students on obvious hazards was shorter than that on hidden hazards (*F* = 13.596, *p* = .000 < .001), and the average fixation duration of intermediate swimming college students with high levels of sensation‐seeking on hidden hazards proved to be significantly shorter (*F* = 5.498, *p* = .000 < .001).

**Conclusions and implications:**

High‐sensation‐seeking intermediate swimming college students exhibited longer reaction times to hidden hazards compared to their low‐sensation‐seeking peers. These findings indicate that a high‐sensation‐seeking tendency can result in delayed reaction times and a disregard for response measures among intermediate swimming college students.

## Graphical abstract

High sensation‐seeking intermediate swimming college students had longer reaction times to hidden hazards than their low sensation‐seeking peers. Our findings indicate that a high sensation‐seeking tendency prolongs the reaction time and leads to the neglect of response measures among intermediate swimming college students.

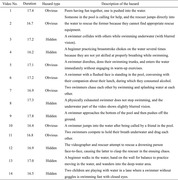

## 1 INTRODUCTION

According to two documents released by the World Health Organization, namely, the *Global report on drowning: preventing a leading killer* in 2014 (World Health Organization, 2014) and *Preventing drowning: an implementation guide* in 2017 (World Health Organization, 2017), approximately 372,000 people drown each year worldwide, equivalent to around 42 individuals per hour. Half of these drowning victims are under the age of 25, with Chinese adolescents aged 18–24 years being the most affected group.

In China, Wang et al. (2018) developed the standard of college students’ water safety skills, which divides college students’ swimming skills into primary, intermediate, and advanced levels. Intermediate swimming college students are defined as individuals who have mastered one or two swimming strokes but lack self‐rescue and drowning rescue skills. Intermediate swimming college students are known to be more prone to risk‐taking behavior and drowning incidents. These students account for 14.6% of the total college student population (Zhang, 2020). Despite having swimming skills, many intermediate swimming college students are not adequately prepared to calmly respond to hazards or effectively assist others in danger. Consequently, their risk of drowning is significantly higher compared to elementary swimming college students, who cannot swim, and advanced swimming college students, who possess self‐rescue and rescue skills. Some intermediate swimming college students may attempt to rescue others in danger out of a sense of virtuousness and courage, only to end up drowning themselves (Zhang, 2020). Research in this field has identified poor hazard perception as the primary internal cause of the high incidence of safety accidents among students (Crundall et al., 2012). Hazard perception involves the process of identifying, evaluating, and responding to potential hazards that could lead to accidents (Habibzadeh et al., 2023). Although studies on the safety of students in water have primarily focused on types and patterns of drowning, educational measures, and rescue techniques, insufficient attention has been given to individual‐level internal causes. Therefore, conducting a systematic analysis of the characteristics of hazard perception among groups at high risk of drowning not only reveals the causes of accidents at an individual level but also enhances accident prevention efforts and facilitates the development of targeted measures at a broader level.

Four indices are commonly used to measure hazard perception: reaction time, subjective assessment, eye movement, and signal detection theory (Asadamraji et al., 2019). Reaction time is measured by asking subjects to rapidly push a button or click a mouse while watching videos that simulate hazardous situations or real videos taken from a first‐person perspective (Zhu & Chang, 2020). Although college students are generally good at visual search, their abilities to identify dangers and levels of hazard perception vary greatly (Malone & Brunken, 2021). While swimming, they can usually respond quickly to hazards within their line of sight, but detecting hidden hazards that are out of sight or have the potential to occur is often challenging (Zhu, 2015). Therefore, the visibility of hazards plays a crucial role in college students’ perception of danger.

Sensation‐seeking is a personality trait characterized by a desire for novel, complex, changeable, intense sensory stimuli, and experiences, often involving willingness to take economic, safety, and legal risks (Egal et al., 2022). Individuals with high levels of sensation‐seeking tend to derive pleasure and satisfaction from engaging in highly risky water‐related activities, such as swimming after consuming alcohol, diving, or swimming in challenging waters (Leavy et al., 2022). College students with high‐sensation‐seeking tendencies often exhibit excessive confidence in their swimming abilities, leading them to overestimate their skills and seek thrilling experiences while disregarding or underestimating the associated risks (Zhang, 2020). As a result, there is a significant positive correlation between sensation‐seeking scores and engaging in highly risky swimming behavior, although the relationship with reaction time to hazards remains unclear.

In summary, this study investigated the correlation between higher levels of sensation‐seeking in college students and an increased risk of drowning. Drawing upon previous theories and utilizing Steinberg et al. (2008) sensation‐seeking scale, we examined the reaction time to hazards and eye movement data of intermediate swimming college students with varying levels of sensation‐seeking. To ensure controlled experimentation, we manipulated the visibility of hazards in the videos. On this basis, the following hypotheses were formulated: (1) H1—intermediate swimming college students with high‐sensation‐seeking levels exhibit longer reaction times to hidden hazards compared to those with low‐sensation‐seeking levels; (2) H2—the duration of the first fixation on obvious hazards is shorter for intermediate swimming college students, and high‐sensation‐seeking students have a shorter average fixation duration on hidden hazards.

## 2 RESEARCH METHODS

### 2.1 Subjects

We selected 400 students from a university in Hubei Province, China who self‐identified as “able to swim” for the preliminary swimming test. Following the college students’ water safety skill standard (Wang et al., 2018), only 283 (197 men and 86 women) qualified as intermediate swimming undergraduates (scores between 60 and 79.9). All of these students were asked to complete a sensation‐seeking scale (Steinberg et al., 2008), and we ranked them from highest to lowest. We invited the top 10% of male participants (20) and 10% of female participants (9) to take the test, but one female participant declined. So, the top 10% consisted of 20 men and 8 women (*M* = 3.13, *SD* = .53) (*t* = 27.516, *p* < .01). The bottom 10% was the same as the top 10%, with one female participant wishing not to participate (*M* = 4.39, *SD* = .32). So, each group consisted of 28 students. All participants were proficient in using a computer mouse and had normal visual acuity (corrected visual acuity). Table 1 provides an overview of the basic information of the subjects in both groups. Statistical analysis revealed no significant differences in the male‐to‐female ratio, years of swimming experience, and scores on the comprehensive swimming assessment between the two groups of college students.

**TABLE 1:** Basic information about the subjects.

Variable	High levels of sensation‐seeking group (*N* = 28)	Low levels of sensation‐seeking group (*N* = 28)
Gender	20 men and 8 women	20 men and 8 women
Age	Aged 19–23, with an average age of 21.43 years	Aged 19–23, with an average age of 20.79 years
Years of swimming experience	3.71	3.62
Comprehensive swimming assessment score	71.47	72.06

### 2.2 Research materials and instruments

#### 2.2.1 College students’ water safety skill standard

This standard examines college students’ swimming skills and rescue skills, with each aspect accounting for 50% of the evaluation. It consists of 38 evaluation indices and is structured into 3 levels (elementary, intermediate, and advanced), and 9 sub‐levels. This comprehensive assessment framework is utilized to evaluate the safety levels of college students’ swimming behavior. The standard has been widely promoted and employed in the evaluation of college students’ swimming skills in China (Table 2), with a strong positive correlation (*r* = .82, *p* < .01).

**TABLE 2:** Assessment standard of intermediate students’ water safety skill grades (Wang et al., 2018).

Level 1 index	Level 2 index	Level 3 index	Band four (60%)		Level five (80%)		Level six (100%)	
			Standard	Score	Standard	Score	Standard	Score
Swimming skill	Breaststroke	25‐m (score) Quality of action (standard) Endurance (standard)	13–22 1 1	7.1 1.6 1.5	23–33 2 2	9.5 2.1 2	≥34 3 3	11.9 2.6 2.5
	Freestyle	25‐m (score) Quality of action (standard) Endurance (standard)	7–12 1 1	4.2 0.9 0.9	13–19 2 2	5.6 1.2 1.2	≥20 3 3	7 1.5 1.5
	Overarm sidestroke	25‐m (score) Quality of action (standard) Endurance (standard)	3–7 1 1	2.5 0.5 0.5	8–10 2 2	3.4 0.7 0.7	≥11 3 3	4.2 0.9 0.9
	Treading water called for help	The number of times your feet touch the bottom of the pool in 1 min (score) Action effectiveness (standard) Endurance (standard)	5–19 1 1	7.1 1.5 1.6	20–31 2 2	9.5 2 2.1	≥32 3 3	11.9 2.5 2.6
Rescue skill	Throw buoying device	3 groups of throw buoying device (score) Action effectiveness (standard)	8–17 1	6.7 2.9	18–28 2	8.9 3.8	≥29 3	11.2 4.8
	Reach for life supplies	3 groups of reach for life supplies (score) Action effectiveness (standard)	9–17 1	6.3 2.7	18–28 2	8.4 3.6	≥29 3	10.5 4.5
	Stretch rescue with bare hands	3 groups of close stretch rescue with bare hands (score) Action effectiveness (standard)	12–25 1	8.0 3.4	26–34 2	10.6 4.6	≥35 3	13.3 5.7

#### 2.2.2 Sensation‐seeking scale

The sensation‐seeking scale used in this study was a revised and translated version developed by Steinberg et al. (2008). The scale consisted of six items: “I love new and exciting experiences, even if they're a little scary,” “I like doing things just because it's exciting,” “I like to do things that are a little scary sometimes,” “I'm going to try anything once,” “I sometimes do crazy things just for fun,” and “I like to be wild.” A six‐point Likert scale was utilized to record the responses: (1) completely out of line with; (2) relatively inconsistent; (3) a little out of line; (4) kind of fit; (5) relative match; (6) fits very well. The sensation‐seeking score for each participant was calculated as the average of their scores on all items. A higher score indicated a higher level of sensation‐seeking. The *α* coefficient of the sensation‐seeking scale used in this study was 0.860.

#### 2.2.3 Testing of college students’ hazard perception

In the test, 16 real videos of students swimming were used. All the videos were shot from a swimmer's perspective using a GoPro Hero9 sports camera in swimming pools. Each video featured a slowly forming potential hazard, which was classified into two categories: obvious hazards and hidden hazards. The classification was based on whether the visibility of the hazard was continuous or not. In the videos, the appearance and development of an obvious hazard were fully visible to the subjects, whereas a hidden hazard was partially or completely concealed from their sight (Sun et al., 2019). For this study, seven videos with obvious hazards, seven with hidden hazards, and two for practice purposes were included. The total length of the hazards captured in the videos was approximately 90 s, and there were no significant differences in the duration of the hazards (*t* = .34, *p* > .05) (Table 3). In terms of time and position, the hazards occurred randomly in the videos. Our experiment had received ethical approval from the biomedical research of Hubei Minzu University (NO. 2020‐45).

**TABLE 3:** Basic information about hazards in the swimming videos.

Video No.	Duration	Hazard type	Description of the hazard
1	17.4	Obvious	Peers having fun together, one is pushed into the water
2	16.7	Obvious	Someone in the pool is calling for help, and the rescuer jumps directly into the water to rescue the former because they cannot find appropriate rescue equipment
3	17.2	Hidden	A swimmer collides with others while swimming underwater (with blurred vision)
4	16.2	Hidden	A beginner practicing breaststroke chokes on the water several times because they are not yet skilled at properly breathing while swimming
5	17.1	Obvious	A swimmer disrobes, dons their swimming trunks, and enters the water immediately without engaging in warm‐up exercises
6	17.2	Obvious	A swimmer with a flushed face is standing in the pool, conversing with their companion about their lunch, during which they consumed alcohol
7	16.9	Obvious	Two swimmers chase each other by swimming and splashing water at each other
8	17.3	Hidden	A physically exhausted swimmer does not stop swimming, and the underwater part of the video shows slightly blurred vision
9	17.8	Hidden	A swimmer approaches the bottom of the pool and then pushes off the ground
10	16.4	Obvious	A swimmer jumps into the water after being called by a friend in the pool
11	16.8	Obvious	Two swimmers compete to hold their breath underwater and drag each other
12	16.9	Hidden	The videographer and rescuer attempt to rescue a drowning person face‐to‐face, causing the latter to clasp the rescuer in the ensuing chaos
13	17.0	Hidden	A beginner walks in the water, hand on the wall for balance to practice moving in the water, and wanders into the deep‐water area
14	16.5	Hidden	Two children are playing with water in a lane where a swimmer without goggles is swimming fast with closed eyes

#### 2.2.4 Eye tracker

The VR eye tracker developed by QingTech Shanghai was used to record subjects’ eye movements at a sampling frequency of 120 Hz. Tobii Studio 3.0 was utilized to define dynamic areas of interest (AOIs), with each AOI in the videos measuring 320 × 200 pixels in size.

### 2.3 Experimental design

A mixed experimental design was employed, utilizing a 2 (sensation‐seeking: high, low) × 2 (hazard type: obvious hazard, hidden hazard) design. Sensation‐seeking, serving as the between‐subjects variable, consisted of two levels: high and low. The hazard type, acting as the within‐subjects variable, was also divided into two types: obvious and hidden.

### 2.4 Experimental procedure

**FIGURE 1:**
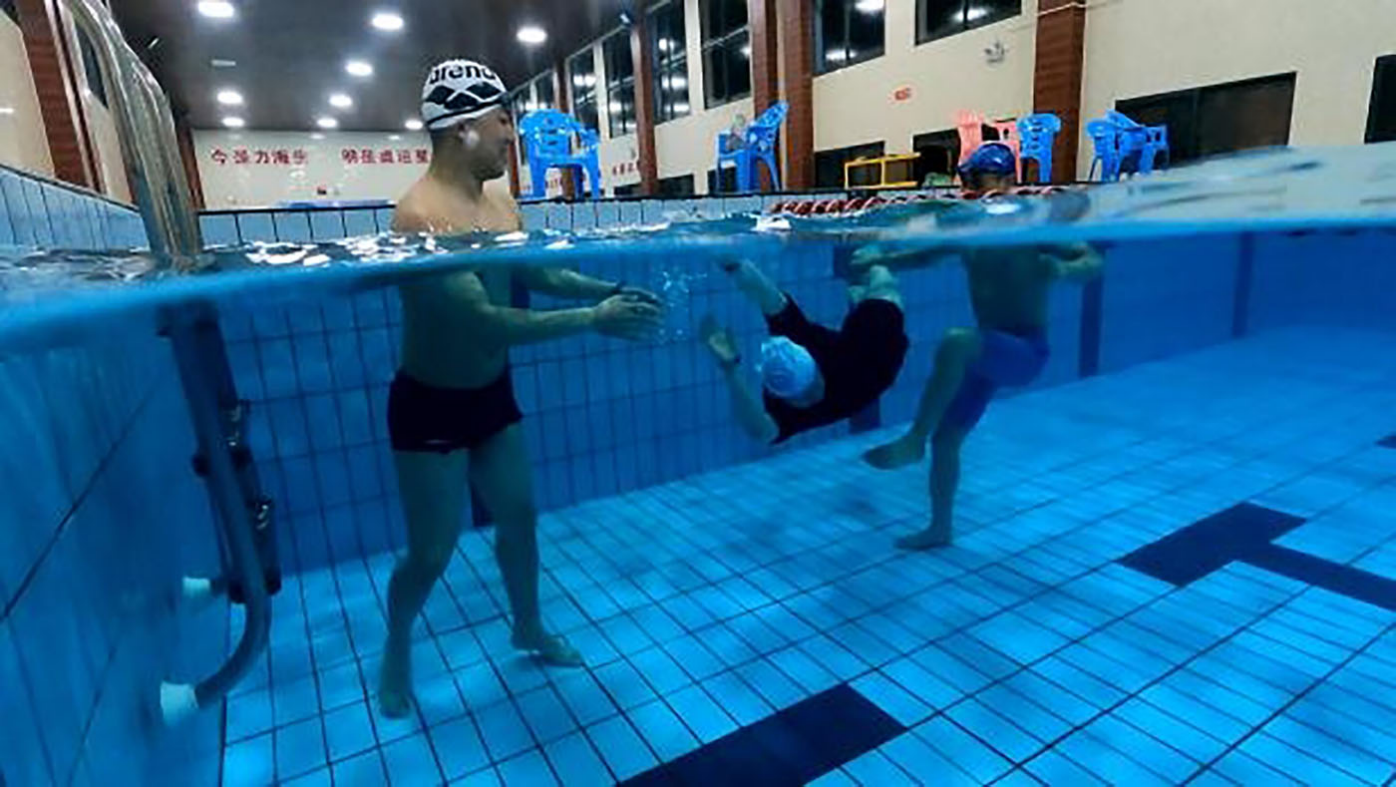
Perspective of the subjects when watching the swimming videos with hazardous situations.

**FIGURE 2:**
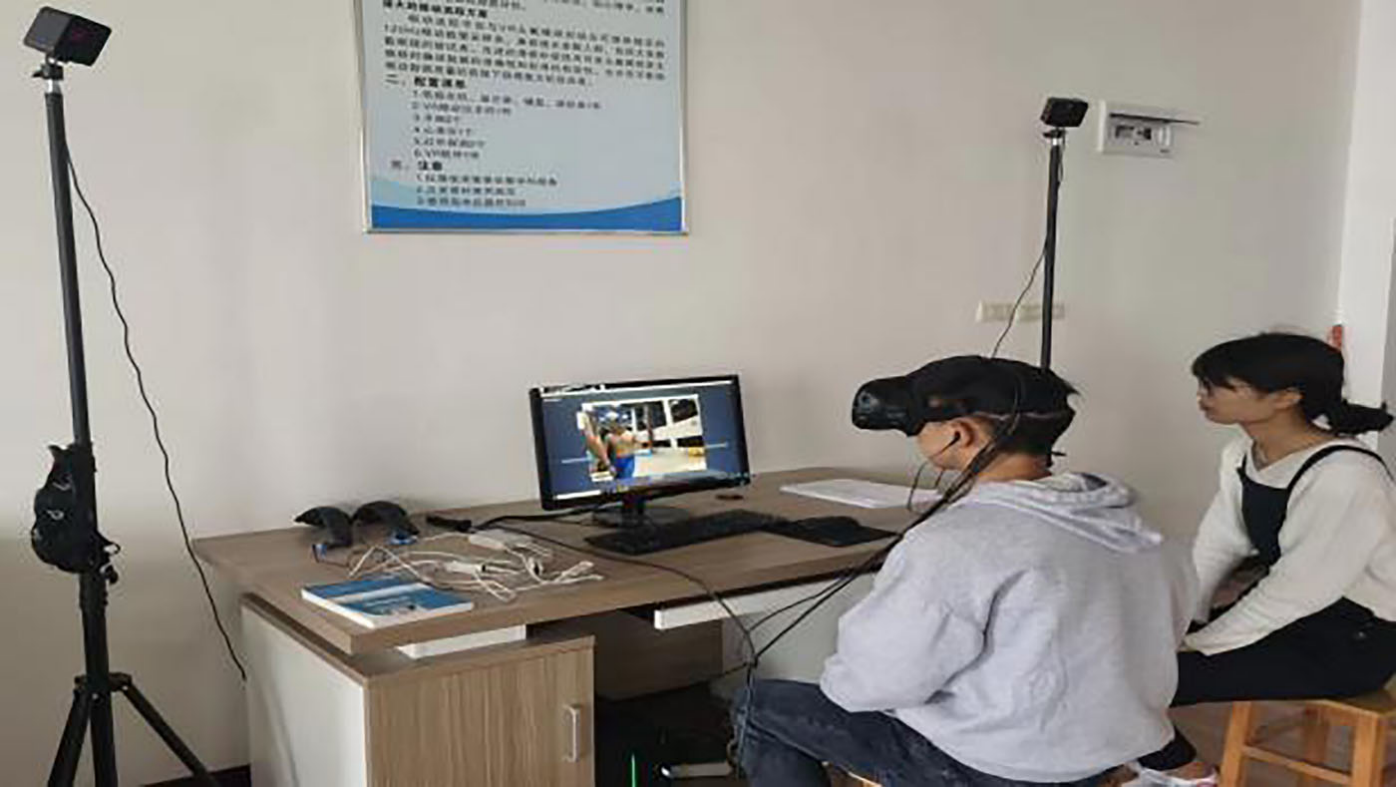
Eye‐tracking.

The subjects were selected, and swimming videos were imported to complete the experimental design. The test assistant explained the procedure and purpose of the experiment, and the subjects filled out the informed consent form and demographic questionnaire, which covered aspects, such as age, gender, and the number of years of swimming. Under the guidance of the experimenter, the subjects donned helmets for the nine‐point calibration. Prior to the formal test, each subject practiced with two videos. Once the experiment started, the subjects watched the videos from the perspectives of the individuals who shot them (Figure 1) and promptly clicked the left mouse button when they noticed potential hazards in the videos (Figure 2). The entire experiment lasted approximately 20 min, and each subject received a Speedo swimming kit after the experiment.

### 2.5 Data statistics and analysis

The test results were recorded using the EyeControl eye‐movement analysis software V2.0 platform developed by QingTech. To analyze the data, SPSS 26.0 software was employed to conduct repeated measures. ANOVA was used for reaction time and eye movement data, including the first fixation duration and average fixation duration. Reaction time refers to the time interval (measured in seconds) between the occurrence of a hazardous situation and the subject clicking the button. The first fixation duration indicates the length of the subject's first fixation on the AOI, providing insight into how quickly a subject can identify a potential hazard. The average fixation duration (measured in seconds) represents the ratio of the total duration of a subject's fixations to the number of fixations within the AOI. It reflects the allocation of attentional resources by a subject in processing a hazard after perceiving it (White et al., 2022). The acquisition rate of the subjects’ eye movements was greater than 80%. Therefore, the eye movement data of all subjects were retained for statistical analysis in this study. The subjects’ reaction time and eye movement data in response to the two types of hazards are presented in Table 4.

**TABLE 4:** Descriptive statistics and multivariate covariance test for reaction time and eye movement data.

			Degree of freedom	Reaction time	First fixation duration	Average fixation duration
Descriptive statistics	Obvious hazard	High‐sensation‐seeking		3.14 ± 0.22	0.28 ± 0.04	0.37 ± 0.04
		Low‐sensation‐seeking		3.04 ± 0.16	0.27 ± 0.05	0.46 ± 0.04
	Hidden hazard	High‐sensation‐seeking		3.65 ± 0.17	0.31 ± 0.03	0.36 ± 0.04
		Low‐sensation‐seeking		3.25 ± 0.32	0.31 ± 0.05	0.39 ± 0.04
Dependent variables	Hazard type		1	0.000^***^	0.000^***^	0.000^***^
	Sensation‐seeking		12	0.000^***^	0.679	0.000^***^
	Hazard type * sensation‐seeking (*p* value)		12	0.001^***^	0.488	0.001^***^
Demographic factors	The main effect of gender (*p* value)			0.858	0.437	0.132
	The main effect of age (*p* value)			0.465	0.095	0234
	The main effect of the years of swimming experience (*p* value)			0.185	0.102	0.847

*Note*:***p<.001.

## 3 RESULTS AND ANALYSIS

To test the effects of demographic variable factors on the dependent variables, a multivariate analysis of covariance was conducted, which revealed significant grouping effects (*F* = 33.025, *p* < .01) and insignificant effects of gender, age, and years of swimming experience.

### 3.1 Reaction time

The repeated measures analysis of reaction time revealed significant findings. First, the main effect of sensation‐seeking was significant (*F* = 6.251, *p* = .000, $\eta_{p}^{2}$ = .475) (partial eta squared refers to partial eta squared values [$\eta_{p}^{2}$] expression). The effect size ranges from 0 to 1, and a larger value indicates a larger difference. An effect size below 0.10 indicates a very small difference, 0.25 indicates a moderate critical point, and an effect size greater than 0.4 indicates a very large difference. In this study, differences greater than 0.25 in effect size were mainly considered, indicating that the reaction time of intermediate swimming college students with low levels of sensation‐seeking was shorter compared to those with high levels of sensation‐seeking. Second, the main effect of hazard type was significant (*F* = 49.438, *p* = .000, $\eta_{p}^{2}$ = .373). This suggests that intermediate swimming college students had a longer reaction time to hidden hazards than to obvious hazards. Furthermore, the interaction effect of sensation‐seeking and hazard type was significant (*F* = 2.647, *p* = .005, $\eta_{p}^{2}$ = .277). Upon conducting a simple effect analysis, we found that intermediate swimming college students with high levels of sensation‐seeking had a longer reaction time to hidden hazards compared to those with low levels of sensation‐seeking (*F* = 68.493, *p* = .000, $\eta_{p}^{2}$ = .388). Additionally, intermediate swimmers with low levels of sensation‐seeking exhibited a shorter reaction time to obvious hazards compared to those with high levels of sensation‐seeking (*F* = 11.396, *p* = 0.001, $\eta_{p}^{2}$ = 0.095).

### 3.2 First fixation duration

The repeated measures analysis of the first fixation duration showed that the main effect of the hazard type was significant (*F* = 13.596, *p* = .000, $\eta_{p}^{2}$ = .141). Specifically, the first fixation duration of intermediate swimmers on obvious hazards was shorter than that on hidden hazards. However, the main effect of sensation‐seeking was not significant (*F* = .172, *p* = .679), and there was no significant interaction effect between sensation‐seeking and the hazard type (*F* = .485, *p* = .488).

### 3.3 Average fixation duration

The repeated measures analysis of average fixation duration showed that the main effect of sensation‐seeking was significant (*F* = 5.498, *p* = .000, $\eta_{p}^{2}$ = .443). Intermediate swimmers with low levels of sensation‐seeking had longer average fixation durations than those with high levels of sensation‐seeking. The main effect of hazard type was also significant (*F* = 5.158, *p* = .026, $\eta_{p}^{2}$ = .059). Intermediate swimming college students’ average fixation duration on obvious hazards was longer than that on hidden hazards. The interaction effect of sensation‐seeking and hazard type also proved significant (*F* = 12.084, *p* = .001, $\eta_{p}^{2}$ = .103). The simple effect analysis of the interaction effect suggested that the group with low levels of sensation‐seeking had a longer average fixation duration on obvious hazards than on hidden hazards (*F* = 39.319, *p* = .000, $\eta_{p}^{2}$ = .267). However, the average fixation duration of the group with high levels of sensation‐seeking did not differ significantly between the two types of hazards (*F* = 1.245, *p* = .268).

## 4 DISCUSSION

In this study, we utilized 16 videos of students swimming as experimental stimuli to investigate the impact of sensation‐seeking and the hazard type on hazard perception among intermediate swimmers. The findings revealed that the hazard type influenced participants’ reaction time, average fixation duration, and first fixation duration. On the other hand, sensation‐seeking only influenced their reaction time and average fixation duration, whereas it had no effect on their first fixation duration. There was an interaction effect between sensation‐seeking and hazard type concerning reaction time and average fixation duration, suggesting that the influence of sensation‐seeking on these measures varied depending on the hazard type.

In terms of reaction time, our findings supported H1, as intermediate swimming college students with high levels of sensation‐seeking exhibited longer reaction times to hidden hazards compared to those with low levels of sensation‐seeking. This can be attributed to the fact that obscured or unpredictable hazards require more time for individuals to identify (Ma et al., 2021). Many studies have shown that unpredictable hazards can significantly increase drivers’ perception of stress, making it too late for the driver to react timely and correctly (Kerautret et al., 2022). In swimming pools specifically, it is often challenging to observe hazards underwater from above the water surface due to factors such as crowdedness and water quality (Hammers & Suhr, 2010). Furthermore, individuals with high‐sensation‐seeking tendencies are prone to engaging in risky swimming behavior and seeking out thrilling challenges, driven by the excitement they experience (Zeng, 2015). This inclination toward excitement further hinders their ability to concentrate on identifying hidden hazards. Our study demonstrated that intermediate swimming college students with low levels of sensation‐seeking exhibited shorter reaction times to obvious hazards compared to those with high levels of sensation‐seeking. This suggests that individuals with high‐sensation‐seeking tendencies require more time for their cognitive control system to identify and respond to hazards. Consequently, they are more inclined to overlook the potential consequences of hazardous factors.

Our results support H2, as intermediate swimming college students with low levels of sensation‐seeking exhibited longer average fixation durations on obvious hazards compared to those with high levels of sensation‐seeking. Interestingly, the group with high‐sensation‐seeking did not show a significant difference in average fixation duration between the two types of hazards. This suggests that both groups of students were able to quickly identify obvious hazards upon viewing them. However, the low‐sensation‐seeking group spent more time analyzing the hazards, as reflected by their longer average fixation duration. In contrast, the high‐sensation‐seeking group had shorter average fixation durations on obvious hazards and was more likely to disregard hazard evaluation. Previous research has shown that adolescents with high levels of sensation‐seeking experience an increase in prefrontal and para‐prefrontal dopamine secretion. This makes them more susceptible to novel, impulsive, and potentially risky impulses (Zhang et al., 2017, 2016) and are more likely to disregard the potential consequences of hazards (Leavy et al., 2022). Sensation‐seeking predicts negative and positive risk‐taking, whereas extroversion and openness are predominantly related to positive risk‐taking (Patterson et al., 2023). Even subconsciously, high‐sensation‐seeking college students completely ignore risks and are able to fully cope with them even when there are risks (Zhang, 2020). This can effectively explain why college students with high levels of sensation‐seeking fail to respond rapidly to hazards in real‐world drowning accidents and may even exhibit excitement and boastfulness in clearly hazardous situations.

In summary, this study examined the hazard perception characteristics of a group faced with a high risk of drowning to explain why college students with higher levels of sensation‐seeking are more prone to drowning. On one hand, intermediate swimming college students with high levels of sensation‐seeking exhibited longer reaction times to water hazards, particularly hidden hazards, compared to those with low levels of sensation‐seeking. On the other hand, both groups of intermediate swimmers were able to identify obvious hazards easily, although the high‐sensation‐seeking group took significantly longer to respond. College students tend to be independent and adventurous, seeking excitement. They are generally helpful when they observe others in trouble. However, they sometimes act impulsively, which poses a significant risk to themselves and others when encountering hazards. This is similar to how professional drivers engage in speeding behavior less often than the general population and young drivers (Sarbescu & Rusu, 2021). Therefore, high‐sensation‐seeking college students are the key population to prevent from drowning when swimming. This study provides scientific support for the development of intervention training programs aimed at improving hazard perception among intermediate swimming college students with high levels of sensation‐seeking.

## 5 CONCLUSION

Hazard perception among intermediate swimming college students with varying levels of sensation‐seeking is influenced by the hazard type. Those with high levels of sensation‐seeking exhibited longer reaction times to hidden hazards compared to those with low levels of sensation‐seeking. The duration of the first fixation duration on obvious hazards was shorter than that on hidden hazards for all intermediate swimming college students. Furthermore, students with high levels of sensation‐seeking had shorter average fixation durations on hidden hazards. These findings suggest that a high‐sensation‐seeking tendency contributes to prolonged reaction times and a tendency to neglect appropriate response measures among intermediate swimming college students.

## 6 LIMITATIONS AND FUTURE RESEARCH

This study makes an important contribution to knowledge and improved understanding of hazard perception among intermediate swimming college students with varying levels of sensation‐seeking by confirming that the said perception is influenced by the hazard type. However, there are several limitations that may impact the results of this study.

First, the subjects in this study were intermediate swimming college students. The influence of different levels of sensation‐seeking and other levels of college students’ risk perception on risk types were not included in the design, and the experimental subjects were limited. Second, primary and secondary school students should enjoy more protection, but this was not covered in our study, and this is the next step to expand the body of knowledge.

In future research, it is recommended to expand the scope of subjects to include primary and middle school students who are also susceptible to drowning. Additionally, further exploration is needed to develop interventions targeting hazard perception among students with high levels of sensation‐seeking. These interventions can play a crucial role in effectively preventing drowning accidents among this specific group of students.

## AUTHOR CONTRIBUTIONS

*Conceptualization; formal analysis; funding acquisition; methodology; resources; software*: Hui Zhang. *Data curation; formal analysis; writing—original draft; writing—review and editing*: Li Zi Yun. *Investigation; supervision; validation*: Shi Luo.

## CONFLICT OF INTEREST STATEMENT

The authors declare no conflicts of interest.

### PEER REVIEW

The peer review history for this article is available at https://publons.com/publon/10.1002/brb3.3338.

## Data Availability

The data used to support the findings of this study are available from the corresponding author upon request.

## References

[brb33338-bib-0001] Asadamraji, M. , Saffarzadeh, M. , Ross, V. , Borujerdian, A. , Ferdosi, T. , & Sheikholeslami, S. (2019). A novel driver hazard perception sensitivity model based on drivers’ characteristics: A simulator study. Traffic Injury Prevention, 20(5), 492–497. 10.1080/15389588.2019.1607971 31180727

[brb33338-bib-0002] Crundall, D. , Chapman, P. , Trawley, S. , Collins, L. , van Loon, E. , Andrews, B. , & Underwood, G. (2012). Some hazards are more attractive than others: Drivers of varying experience respond differently to different types of hazard. Accident Analysis & Prevention, 45, 600–609. 10.1016/j.aap.2011.09.049 22269547

[brb33338-bib-0003] Egal, A. , Donon, C. , Jakubiec, L. , Lambert, L. , Fatseas, M. , & Auriacombe, M. (2022). Ordalie, sensation‐seeking and impulsivity. Critical analysis of definitions. Encephale, 48(2), 163–170. 10.1016/j.encep.2021.02.016 34099245

[brb33338-bib-0004] Habibzadeh, Y. , Yarmohammadian, M. H. , & Sadeghi‐Bazargani, H. (2023). Driving hazard perception components: A systematic review and meta‐analysis. Bulletin of Emergency and Trauma, 11(1), 1–12. 36818054 10.30476/beat.2023.95410.1356 PMC9923031

[brb33338-bib-0005] Hammers, D. B. , & Suhr, J. A. (2010). Neuropsychological, impulsive personality, and cerebral oxygenation correlates of undergraduate polysubstance use. Journal of Clinical and Experimental Neuropsychology, 32(6), 599–609. 10.1080/13803390903379599 19937505

[brb33338-bib-0007] Kerautret, L. , Dabic, S. , & Navarro, J. (2022). Detecting driver stress and hazard anticipation using real‐time cardiac measurement: A simulator study. Brain and Behavior, 12(2), e2424. 10.1002/brb3.2424 35092145 PMC8865166

[brb33338-bib-0008] Leavy, J. E. , Della Bona, M. , Abercromby, M. , & Crawford, G. (2022). Drinking and swimming around waterways: The role of alcohol, sensation‐seeking, peer influence and risk in young people. PLoS ONE, 17(11), e0276558. 10.1371/journal.pone.0276558 36331939 PMC9635690

[brb33338-bib-0009] Ma, Y. , Luo, S. , Zhang, H. , & Wang, B. (2021). Investigation on high‐risk swimming behavior and its influencing factors of primary and middle school students. Journal of Adult Physical Education, 37(01), 90–94. 10.16419/j.cnki.42-1684/g8.2021.01.015

[brb33338-bib-0010] Malone, S. , & Brünken, R. (2021). Hazard perception, presence, and simulation sickness—A comparison of desktop and head‐mounted display for driving simulation. Frontiers in Psychology, 12, 647723. 10.3389/fpsyg.2021.647723 33967907 PMC8100057

[brb33338-bib-0011] Patterson, M. W. , Pivnick, L. , Mann, F. D. , Grotzinger, A. D. , Monahan, K. C. , Steinberg, L. D. , Oosterhoff, B. , Tackett, J. L. , Tucker‐Drob, E. M. , & Harden, K. P. (2023). A mixed‐methods approach to refining and measuring the construct of positive risk‐taking in adolescence. Journal of Research on Adolescence, 33(2), 680–700. 10.1111/jora.12807 36358015 PMC10464509

[brb33338-bib-0012] Sârbescu, P. , & Rusu, A. (2021). Personality predictors of speeding: Anger‐aggression and impulsive‐sensation seeking. A systematic review and meta‐analysis. Journal of Safety Research, 77, 86–98. 10.1016/j.jsr.2021.02.004 34092331

[brb33338-bib-0013] Steinberg, L. , Albert, D. , Cauffman, E. , Banich, M. , Graham, S. , & Woolard, J. (2008). Age differences in sensation seeking and impulsivity as indexed by behavior and self‐report: Evidence for a dual systems model. Developmental Psychology, 44(6), 1764–1778. 10.1037/a0012955 18999337

[brb33338-bib-0014] Sun, L. , Ma, Y. , & Chang, R. (2019). Effects of self‐assessed ability and hazard type on young novice drivers’ hazard perception. Journal of Transportation Systems Engineering & Information Technology, 19(1), 228–232. http://www.tseit.org.cn/EN/abstract/abstract19790.shtml

[brb33338-bib-0015] Wang, B. , Yu, H. , Luo, S. , & Zhang, H. (2018). Development of water safety skills grade standard for college students. Journal of Wuhan Institute of Physical Education, 52(3), 89–95. http://open.oriprobe.com/articles/54192538/Development_of_Water_Safety_Skills_Grade_Standard_.htm

[brb33338-bib-0016] White, H. , Heck, A. , Jubran, R. , Chroust, A. , & Bhatt, R. S. (2022). Average fixation duration in infancy: Stability and predictive utility. Infancy, 27(5), 866–886. 10.1111/infa.12483 35624554

[brb33338-bib-0017] World Health Organization . (2014). Global report on drowning: Preventing a leading killer. World Health Organization. Retrieved from https://www.who.int/publications/i/item/global‐report‐on‐drowning‐preventing‐a‐leading‐killer

[brb33338-bib-0018] World Health Organization . (2017). Preventing drowning: An implementation guide. World Health Organization. Retrieved from https://www.who.int/publications/i/item/9789241511933

[brb33338-bib-0019] Zeng, J. (2015). The effect of unconscious ego‐depletion on risk decision‐making [Dissertation, Fujian Normal University].

[brb33338-bib-0020] Zhang, H. (2020). On the layered education model of water safety for college students. China Social Sciences Press. https://www.sklib.cn/booklib/bookpreview?sitelD=122&ID=8167933&fromSublD=297

[brb33338-bib-0021] Zhang, H. , Wang, B. , Luo, S. , & Xia, W. (2017). Exploring factors influencing students water high‐risk practices based on grounded theory. China Safety Science Journal, 27(3), 7–12. 10.16265/j.cnki.issn1003-3033.2017.03.002

[brb33338-bib-0022] Zhang, H. , Wang, B. , Luo, S. , Yu, H. , Fang, Z. , & Bu, S. (2016). Survey on drowning high‐risk behavior among college students in Hubei Province. Hubei Sports Science, 35(4), 300–303.

[brb33338-bib-0023] Zhu, L. (2015). Study on the brain mechanism of the influence of swimming exercise on the emotional state of stressed people [Dissertation, Tianjin University of Sport].

[brb33338-bib-0024] Zhu, P. , & Chang, R. (2020). Effects of gender and situational hazard level on pedestrian hazard perception: Evidence from ERP. Studies of Psychology & Behavior, 18(1), 113–120. 10.1016/j.neulet.2019.134546 31629775

